# Polymorphism in Cannabinol Piperazine Cocrystals: Structural, Morphological, and Energetic Perspectives

**DOI:** 10.1021/acs.cgd.5c01390

**Published:** 2025-11-25

**Authors:** Adéla Koryt’áková, Argyro Chatziadi, Jan Rohlíček, Eliška Zmeškalová, Josef Beránek, Miroslav Šoóš

**Affiliations:** † Department of Chemical Engineering, 534467 University of Chemistry and Technology in Prague, Technická 3, 16628 Prague 6, Czech Republic; ‡ Institute of Physics of the Czech Academy of Sciences, Na Slovance 2, 182 00 Prague 8, Czech Republic; § 537141 Zentiva k.s., U Kabelovny 130, 10237 Prague 10, Czech Republic

## Abstract

Polymorphism remains a major challenge in the development of pharmaceutical solid products as even small changes in the crystal arrangement can influence key properties such as stability and solubility. In this study, we mechanochemically prepared a novel cannabinol piperazine cocrystal, which exists in three polymorphic forms. The formation of these polymorphs was systematically investigated by varying the solvents, temperature, and milling time during the ball mill experiments. Interestingly, the phenomenon of disappearing polymorphs was observed under repeated milling. To get insights into the polymorphic behavior and assess the relative stability of the forms, we analyzed their crystal structures, morphologies, and hydrogen bond motifs and performed particle energy calculations using density functional theory. The theoretical results show good correlation with the experimental data and provide valuable and deep insights into the polymorphic behavior and the disappearance of the metastable form. Overall, this work highlights the importance of integrating structural analysis with energetic evaluations to rationalize and predict polymorph stability and transformations.

## Graphical abstract

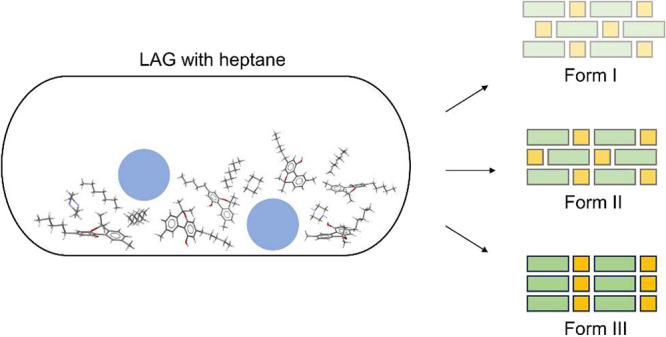

## 1 Introduction

Polymorphism, which is defined as the ability of a compound to crystallize in more than one crystal structure, is a common phenomenon in solid-state chemistry. In pharmaceutical development, polymorphism plays a crucial role because it directly affects key physicochemical properties of solid forms, including stability, solubility, and thermal properties.^1–6^ While multiple polymorphic forms are often observed, their experimental availability is not always straightforward. In some cases, a previously obtained polymorphic form cannot be reproduced under identical or similar crystallization conditions. These cases, widely referred to as “disappearing polymorphs”,^7–9^ highlight the sensitivity of the crystallization processes to factors such as impurities, seeding effect, or competitive nucleation. From a pharmaceutical point of view, disappearing polymorphs present a significant challenge, as they complicate reproducibility and control of the desired polymorphic form.^6,9^ Nevertheless, in the literature, we can also find examples, where these disappeared polymorphic forms have been successfully reproduced under controlled conditions, such as adjusting particle size or using virgin glassware.^9–12^ Thus, understanding and controlling polymorphism is essential for manufacturability of pharmaceutical compounds and also for achieving consistent therapeutic effects.

The polymorphism is not limited only to the single-component solids, but it has also been observed in multicomponent systems.^13–17^ Cocrystals, a well-known system in the pharmaceutical area, consist of an active pharmaceutical ingredient (API) and one or more pharmaceutically acceptable coformers in a defined stoichiometric ratio.^18–20^ They are usually stabilized through noncovalent interactions, such as hydrogen bonding, π–π stacking, or van der Waals forces. The formation of cocrystals enables a fine-tuning of the physicochemical properties, including solubility, thermal stability, mechanical properties, etc.^19,21^ However, polymorphic behavior in cocrystals remains less studied compared to single-component systems, partly due to the limitations of conventional solution-based screening strategies, especially when the API and coformer solubilities differ significantly.^22^

As an alternative way, mechanochemistry has recently emerged with considerable attention as a sustainable, efficient, and green approach for the synthesis of novel solid forms.^23,24^ Among these methods, ball milling is the most commonly used and allows preparation under either neat grinding (NG) or liquid-assisted grinding (LAG) conditions.^25–27^ Contrary to the traditional solution-based methods,^13^ ball milling operates under nonequilibrium conditions, which facilitate the formation of kinetically metastable phases.^25,28,29^ The formation of the metastable forms is highly dependent on many parameters, such as the presence of additives,^2,30^ used frequency,^31,32^ temperature^25,33^ or the material of the milling jars.^34–36^

Cannabinol (CBN) is a nonpsychoactive phytocannabinoid, naturally found in *Cannabis sativa*, alongside more widely studied cannabinoids such as tetrahydrocannabinol (THC) and cannabidiol (CBD).^37,38^ CBN has attracted attention due to its potential therapeutic properties, including anticancer,^39^ anti-inflammatory,^40^ and analgesic effects.^41^ Similar to CBD, cannabinol suffers from poor aqueous solubility and limited stability, which do not enable its direct pharmaceutical applications.^38^ The patent literature^42,43^ has already described four CBN cocrystals, namely, betaine, d-proline, l-proline, and tetramethylpyrazine, which highlights the potential of cocrystallization strategies to optimize CBN physicochemical properties.

In this study, we present a novel unexplored CBN cocrystal with piperazine (CBN-PI), which exhibits rich polymorphic behavior. Three polymorphic forms were obtained through ball milling, and their formations were systematically investigated using a combination of experimental and computational approaches. We employed a combination of experimental and computational approaches to understand their relative stability, crystallographic features, and transformation pathways. In particular, density functional theory (DFT)-based particle energy calculations were used to evaluate the influence of lattice energy, conformational strain, and surface attachment energies on the observed polymorphic landscape. The results provide new insights into the stability of the CBN-PI cocrystal polymorphs and offer a rational explanation for the experimentally observed disappearance of a specific polymorphic form.

## 2 Materials and Methods

### 2.1 Materials

Cannabinol was purchased from Pharmabinoid B.V. (Uden, Netherlands). Piperazine was purchased from Sigma-Aldrich (St. Louis, USA) and was used as received with a purity higher than 98%. Solvents such as acetone (AE), butyl acetate (BA), dichloromethane (DCM), heptane (HP), methanol (MeOH), and tetrahydrofuran (THF) were purchased from PENTA (Prague, Czech Republic).

### 2.2 Milling Experiments

All grinding experiments were carried out with a Retsch MM500 mixer mill. Approximately 50 mg of CBN and piperazine were mixed in a 2:1 molar ratio in a 2 mL polypropylene milling jar with two 5 mm stainless steel balls. All experiments were carried out at a frequency of 25 Hz. Initial screening experiments were carried out for 20 min using heptane as a LAG additive in an amount of 10 μL. Subsequent experiments focused on studying different milling conditions. Various liquid additives (AE, BA, DCM, HP, MeOH, THF, water) were tested in an amount of 10 μL. The milling times varied from 5 to 60 min at room temperature, and in a separate set of experiments, the milling time was fixed at 20 min, while the temperature was adjusted to 5, 25, and 35 °C.

### 2.3 Single-Crystal Preparation

The single crystals of CBN-PI Form I and Form III were prepared by combining CBN and piperazine in a cyclohexane solution in a 2:1 ratio. The mixtures were heated to 35 °C to dissolve the powder and then allowed to cool, followed by slow evaporation until the single crystals were formed. The resulting single crystals were subsequently analyzed according to the conditions that are described below. The preparation of both single crystals was straightforward, in contrast to Form II. Since Form II is not the most stable polymorph, we conducted several seeding experiments from different solvents (cyclohexane, HP, hexane); however, these experiments resulted in the formation of Form III. For this reason, we employed XRPD to solve the crystal structure from powder data.

### 2.4 Characterization

#### 2.4.1 Powder X-ray Diffraction (XRPD)

X-ray diffraction patterns were obtained for both the raw powders and the prepared materials using a powder diffractometer X’Pert^3^ Powder (PANanalytical, Holland) equipped with Cu anode Kα (λ = 1.542 Å) with a tube voltage of 40 kV and a tube current of 30 mA. The data were collected from 4 to 40° 2θ with 0.026° 2θ step size and 56.87 s per step.

#### 2.4.2 Single-Crystal and Powder X-ray Diffraction for the Structure Solution

Single-crystal X-ray diffraction (SCXRD) measurement was performed using two four-circle CCD Rigaku diffractometers: Supernova, equipped with a microfocus Mo X-ray tube and a CCD detector Atlas S2 (Form I, Pc), and XtaLAB Synergy R, equipped with a rotating anode Cu X-ray tube and a HyPix-Arc 150 detector (Form III, P2_1_/n). Both diffractometers use a Cryojet chiller.

The data reduction and absorption correction were done with CrysAlisPro software.^44^ The structures were solved by charge flipping methods using Superflip software and refined by full matrix least-squares on squared value using Crystals^45^ and Jana2020 software.^46^ MCE software^47^ was used for the visualization of residual electron density maps. All H atoms were placed from the residual electron density map, and the C–H atoms were constrained to ideal geometries. The structure of Form I is slightly disordered, with one of the CBD side chains being in two positions with occupancies of 0.828 and 0.172. The sample CBN-PI Form II was ground and placed in a 0.5 mm borosilicate-glass capillary. Powder diffraction data were collected using the Debye–Scherrer transmission configuration on the powder diffractometer Empyrean of PANalytical (λCu,Kα = 1.54184 Å) that was equipped with a focusing mirror, a capillary holder, and a PIXcel^3D^ detector. The data was collected from 3° to 80° 2θ, with a step of 0.013° and with an overall 20 h measurement time.

The structures were deposited into the Cambridge Structural Database under numbers 2483132 (CBN-PI Form I), 2483133 (CBN-PI Form II), and 2483134 (CBN-PI Form III). Details of crystal structure solutions are in the Supporting Information in Section SI 1.

#### 2.4.3 Thermal Analysis

Thermal properties were analyzed using differential scanning calorimetry DSC 3+ (Mettler Toledo, Switzerland) and thermogravimetric analysis (TGA). For DSC measurements, approximately 2 mg of the sample was placed into an aluminum pan, which was sealed and pierced to allow possible solvent vapor to escape and prevent an explosion. The temperature range for DSC experiments was from 20 °C to the specific degradation temperature of each sample with a heating rate of 10 °C/min. For TGA experiments, the pan was filled with approximately 5 mg of the sample and heated from 30 to 300 °C at a rate of 5 °C/min. All measurements were carried out under an inert nitrogen atmosphere.

#### 2.4.4 Solution and Solid-State Nuclear Magnetic Resonance (NMR)

Solution NMR was employed to determine the stoichiometry and purity of the prepared materials. Each sample was dissolved in *d*_6_-DMSO, and the ^1^H NMR spectra were measured with an Avance III 500 MHz NMR spectrometer (Bruker, USA) equipped with a Prodigy probe and with a repetition delay of 10 s.

Solid-state NMR was utilized to confirm cocrystal formation and purity of the obtained material. The ^13^C NMR spectra were measured by an Avance III 400 MHz NMR spectrometer (Bruker, USA) equipped with a 4 mm probe and with 13 kHz spinning.

### 2.5 Computational Procedures

#### 2.5.1 Interaction Energy Calculations and Energy Frameworks

Interaction energies and energy frameworks were calculated using the software CrystalExplorer17 (version 17.5, revision f4e298a).^48^ Molecular wave functions were generated using the built-in Tonto utility in the “accurate” mode using the B3LYP/6-31G­(d,p) level of theory. Visualizations of the energy frameworks were created by using the same software.

#### 2.5.2 Surface and Morphology Analysis

The morphology of each polymorph surface was calculated using the CSD-Particle tool,^49,50^ which is a part of the Mercury software (2025.1.1, build 448738).^51^ The Dreinding II force field with a limiting radius of 30 Å was used for the calculation of the lattice energies and crystal shape with the VisualHabit model, where the lattice energy value can also be found. The surface morphology and topology were calculated by using the Surface Analysis tool. The analysis was focused on the largest predicted facet for each form. The surface offset was automatically selected by the software to be the smoothest one. The examined descriptors included hydrogen bond (HB) acceptors, HB donors, and aromatic bonds.

#### 2.5.3 Calculation of Hydrogen Bonding Propensity and Full Interaction Maps

The hydrogen bonding propensity (HBP) and hydrogen bond coordination (HBC) analyses^52^ were performed using the CSD-Material tool,^53^ a part of Mercury software (4.3.0, build 270015). For the analyses, the default donor/acceptor definitions (O–H and N–H donors and N and O acceptors) and a 0.25 Å heavy-atom distance tolerance were used. For each polymorph, the top-scoring hydrogen bond networks were enumerated and plotted on the HBP/HBC landscape, and the experimentally observed network for each form was overlaid. Full interaction maps (FIMs) were generated with default NH and OH donor probes and N/O acceptor probes for the isolated component molecules. The experimental packings were then inspected against the predicted hotspots. No user retraining or reweighting of the statistical model was applied.

#### 2.5.4 Calculation of Conformational Strain Energy

Conformational strain energies were calculated using DFT in Gaussian 16, applying the B3LYP functional with a D3BJ dispersion correction and the 6-311G­(d,p) basis set. For each polymorph, a single molecule of CBN was extracted from the crystal structure using Mercury. A constrained geometry optimization was performed, where torsion angles were frozen to retain the crystal conformation, while bond lengths and angles were allowed to relax. A fully relaxed gas-phase optimization of the same molecule (with no constraints) was then performed using the same level of theory.^54^ The conformational strain energy was calculated using eq 1:

$$\Delta E_{\mathrm{strain}}=E_{\mathrm{crystal}}-E_{\mathrm{gas}\;\mathrm{phase}}$$ 1

where the first term corresponds to the crystal energy of the constrained structure and the second term is the fully optimized gas-phase structure. Energies were obtained in Hartree and converted to kJ mol^–1^ using the factor 1 hartree = 2625.5 kJ mol^–1^.

#### 2.5.5 Particle Energy Calculations

The particle energy was calculated using eq 2:^10^

$$E_{\mathrm{particle}}=E_{\mathrm{lattice}(\mathrm{inter})}+\Delta E_{\mathrm{conformational}(\mathrm{intra})}-0.5\sum x_{\left(hkl\right)}\times E_{\left(hkl\right)}$$ 2

The first contribution in the equation corresponds to the lattice energy, which describes the strength of intermolecular interactions that hold the molecules together in the bulk. The second term is the conformational energy penalty, representing the energy cost of a molecule to adopt the specific conformation required within the crystal lattice from the lowest energy reference state. The last term is the surface energy penalty, describing how surface termination influences particle stability. It includes the fractional surface area of each crystal face, with the corresponding attachment energy values.

## 3 Results and Discussion

Mechanochemical preparation of pharmaceutical cocrystals is known to be sensitive to both kinetic and thermodynamic factors, often giving rise to polymorphic diversity. In this study, initial screening milling experiments using heptane as a LAG additive resulted in the formation of a CBN-PI cocrystal, in a 2:1 ratio, referred to as Form I. However, subsequent repetitions of the same milling conditions unexpectedly resulted in the appearance of a new polymorph, Form II, followed by the emergence of a third form, Form III, under identical milling parameters, some months later. All polymorphs were initially obtained in their pure forms. Notably, after the appearance of Form III, Form I could not be obtained anymore despite repeating the experiments under the same previously successful conditions. The XRPD patterns of all three polymorphs are shown in Figure 1, confirming distinct phases.

**Figure 1:**
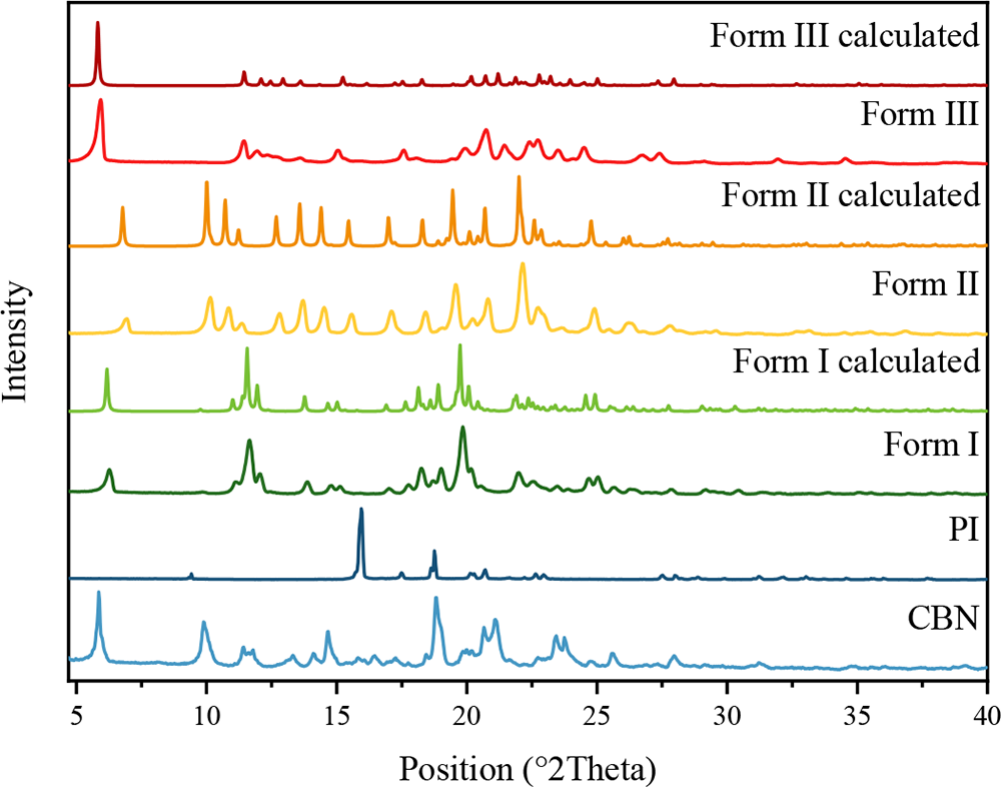
Experimental and calculated patterns of the CBN-PI cocrystal polymorphs I–III compared with their starting materials.

### 3.1 Characterization of the Obtained CBN-PI Polymorphs

To evaluate the thermal behavior and stability of the obtained polymorphs, their thermodynamic properties were investigated using TGA and DSC. The TGA curves (Figure 2a) showed comparable behavior among all polymorphs. All of them show a weight loss of around 13.5% near 150 °C, which is in accordance with the calculated values of 12.2% of the weight loss in a 2:1 ratio, which is connected to the sublimation of the piperazine within the cocrystal lattice.^55^ The DSC thermographs (Figure 2b) show a single sharp endothermic peak for each polymorph, which indicates their phase purity. The melting points of the polymorphs lie between those of the starting materials: CBN shows a melting peak at 76 °C (*T*_onset_ = 74 °C) and a pure piperazine peak at 113 °C (*T*_onset_ = 111 °C). The CBN-PI Form I melts at 91 °C (*T*_onset_ = 88 °C), Form II melts at 95 °C (*T*_onset_ = 91 °C), and Form III melts at 87 °C (*T*_onset_ = 84 °C). Notably, Form I, which was initially obtained but could not be reproduced in later experiments, does not exhibit the lowest melting point among the three polymorphs, as might be expected.^11,56^ However, the stability of the polymorph is determined by more than just its melting point, encompassing parameters such as lattice energy, symmetry, hydrogen bonding network, etc.^55,57^

**Figure 2:**
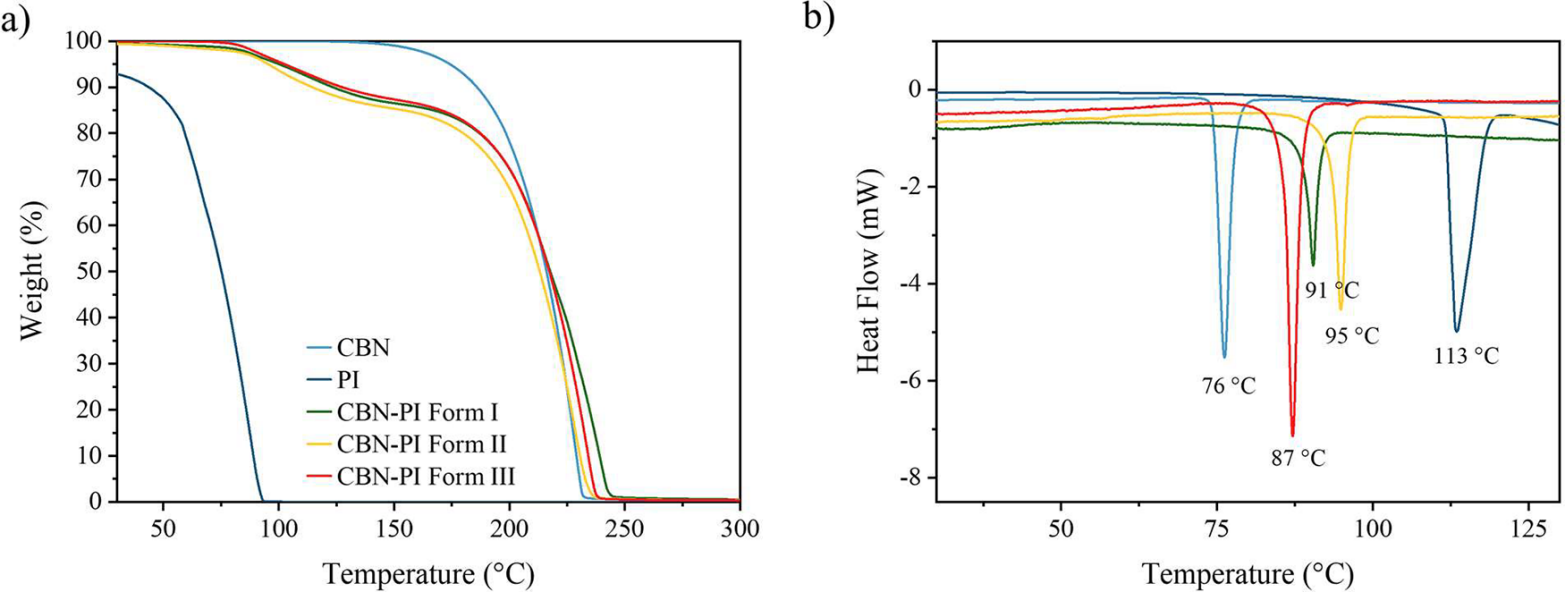
(a) TGA thermograms and (b) DSC thermograms of CBN polymorphs with their starting materials.

The ^1^H NMR spectroscopy confirmed a 2:1 molar ratio in the prepared polymorphic forms. Solid-state NMR was used to confirm the cocrystal formation in the CBN-PI samples and to reveal distinct chemical shift differences, which confirms that each sample represents a unique polymorph. The ssNMR spectra are shown in Figure SI 2.

To better understand and control the formation of each polymorph, a systematic screening was conducted. This included evaluating the effect of different solvents, investigating the influence of milling time, and finally investigating the effect of the milling temperature.

### 3.2 Influence of Different Experimental Conditions on the Polymorphic Form Obtained

#### 3.2.1 Influence of the Solvent

In these experiments, seven different solvents were selected along with NG as a solvent-free condition. The chosen solvents represent a range of commonly used liquid additives in mechanochemistry and also include a variety of functional groups with different polarities. The results are shown in Table 1. The corresponding XRPD patterns are presented in Figure SI 3. Milling in the presence of BA, DCM, and HP resulted in the complete formation of Form III. In contrast, milling experiments with AE and water resulted in partial conversion of the cocrystal in Form III, alongside still present unreacted starting materials. However, the presence of MeOH caused the appearance of a mixture of Forms II and III. Finally, using neat grinding conditions led to partial conversion to Form II in the presence of starting materials. Interestingly, the use of THF did not lead to any cocrystal transformation, and the XRPD pattern corresponds to the physical mixture of pure CBN and PI. Overall, the results reveal a correlation between the solvent polarity and the outcome of the milling experiments. Weakly polar or nonpolar solvents (BA, DCM, HP) do not form any hydrogen bonds with the starting components and lead directly to the interaction between CBN and PI, thus promoting rapid nucleation and growth of the most stable Form III. In contrast, polar liquid additives (AE, MeOH, THF, water) can compete for the hydrogen bonds with the reactants, forming solvated layers or sticky pastes, or even dissolve part of the reactants. These effects reduce or inhibit the efficiency of the nucleation and might stabilize a mixture with a metastable form.^58^

**Table 1:** Polymorphic Forms Obtained at Different Temperatures Using Various Solvents and Neat Grinding Conditions

solvent	polymorphic form
AE	CBN + PI + Form III
BA	Form III
DCM	Form III
HP	Form III
MeOH	Form II + Form III
H_2_O	CBN + PI + Form III
THF	CBN + PI
NG	CBN + PI + Form II

In conclusion, the results show that the choice of solvents influences the efficiency of cocrystal formation based on the solvent polarity. In most cases, Form III tends to be the dominant polymorph. Form II was obtained only under solvent-free conditions, together with the starting materials, and as a mixture with Form III, when milling with MeOH. Based on these findings, we have selected HP, MeOH, and NG for subsequent experiments, as they provide different results and influence the polymorphic forms. Moreover, they represent a diverse set of solvents, including both a nonpolar and a polar solvent and a solvent-free approach.

#### 3.2.2 Influence of Temperature

Another factor that was systematically studied was the influence of milling temperature. Experiments were performed at 5, 25, and 35 °C to simulate varying conditions that might affect polymorphic formation. The results are reported in Table 2, and XRPD patterns are shown in Figure SI 4. Milling in the presence of methanol and heptane led to the formation of pure Form III across all three temperatures except at room temperature with MeOH, where a mixture of Forms II and III was obtained. Interestingly, under NG conditions, incomplete conversion of Form II was observed only at room temperature, while at 5 and 35 °C, the incomplete formation of Form III was detected. These results indicate that temperature has no significant effect on the polymorphic formation under these solvent conditions and highlight the dominance of Form III.

**Table 2:** Polymorphic Forms Obtained at Different Temperatures Using Methanol, Heptane, and Neat Grinding Conditions

	5 °C	25 °C	35 °C
HP	Form III	Form III	Form III
MeOH	Form III	Form II + III	Form III
NG	CBN + PI + Form III	CBN + PI + Form II	CBN + PI + Form III

#### 3.2.3 Influence of Time

Lastly, the effect of milling time on polymorphic formation was investigated. The experiments were carried out using heptane, methanol, and under NG conditions with milling durations ranging from 5 to 60 min. The obtained polymorphic forms are summarized in Table 3, and the corresponding XRPD patterns are presented in Figure SI 5. When heptane was used, the pure polymorphic form III was obtained across all time points. In contrast, milling with methanol led to the consistent formation of a mixture of Forms II and III throughout the entire time. Under NG conditions, no full cocrystal conversion of Form II was observed at any time point. These findings show that the use of a liquid additive facilitates full conversion of the cocrystal. Furthermore, the type of the solvent plays a key role in the resulting polymorphic form, favoring specific Form III or stabilizing a mixture of Forms II and III.

**Table 3:** Polymorphic Forms Obtained at Different Time Points Using Methanol, Heptane, and Neat Grinding Conditions

	5 min	10 min	15 min	20 min	30 min	40 min	60 min
HP	Form III	Form III	Form III	Form III	Form III	Form III	Form III
MeOH	Form II + III	Form II + III	Form II + III	Form II + III	Form II + III	Form II + III	Form II + III
NG	CBN + PI + Form II	CBN + PI + Form II	CBN + PI + Form II	CBN + PI + Form II	CBN + PI + Form II	CBN + PI + Form II	CBN + PI + Form II

In conclusion, despite a systematic investigation of various experimental parameters, including solvent, temperature, and milling time, it was not possible to selectively obtain each polymorphic form under specific conditions. Moreover, the Form I could not be reproduced under any of the tested conditions, which suggests the occurrence of the well-known “disappearing polymorph” phenomenon.^7,8,11^ Form II was obtained only as a minor phase, either in mixtures with Form III or along with incompletely converted starting materials. Interestingly, in most cases, Form III was the dominant polymorphic form, indicating its strong preference as the thermodynamically most stable form despite having a slightly lower melting point compared to that of Forms I and II.

### 3.3 Crystal Structure Characterization

To further understand the observed polymorphic behavior of the CBN-PI cocrystal polymorphs, we determined the crystal structures of all prepared polymorphs. Crystallographic data and details of the structure refinement are listed in Section S1. Furthermore, we calculated the lattice energies and intermolecular interaction energies to evaluate the packing efficiency and stability of each polymorph.

#### 3.3.1 CBN

The crystal structure of pure CBN has been known since 1977.^59^ It crystallizes in the monoclinic space group *P* 2_1_/*c*. The asymmetric unit (Figure SI 6a) consists of two molecules of CBN, while in the unit cell (Figure 3a), there are eight molecules of CBN. The two unique molecules in the asymmetric unit are connected by a hydrogen bond via oxygen atoms (Figure SI 6b). The molecules of CBN form infinite chains linked by hydrogen bonds within each chain. The calculated crystal shape (Figure 3b) shows that the largest facets are (100) and (−100), which are equal and parallel, and together account in total 44.75% of the crystal surface area. The CBN chains create channels in which the slip planes are oriented and align with the direction of the largest facets of the crystal. The calculated lattice energy for this crystal is −180.0 kJ mol^–1^. The strongest calculated interaction (−48.5 kJ mol^–1^) is between two unique CBN molecules (Figure SI 6c), which correspond to the described hydrogen bonding motif.

**Figure 3:**
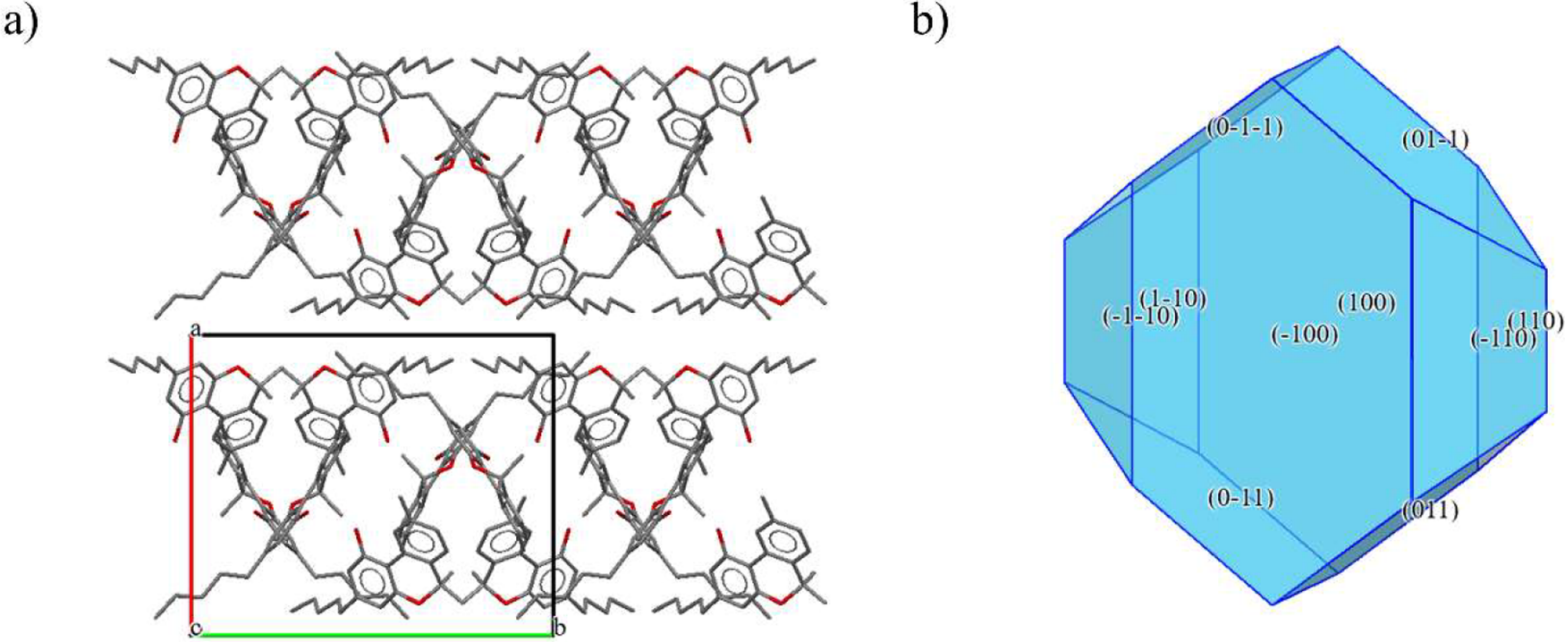
Crystal structure of CBN: (a) unit cell and (b) calculated crystal shape.

#### 3.3.2 CBN-PI Form I

CBN-PI Form I crystallizes in the monoclinic system with space group Pc. In the asymmetric unit (Figure 4a), there are two unique molecules of CBN and one piperazine molecule, with one CBN molecule exhibiting disorder in its aliphatic chain. The unit cell (Figure SI 7a) comprises eight CBN and four PI molecules. The hydrogen bonding network (Figure SI 7b) is relatively complex: one PI molecule connects to three molecules of CBN. Both N atoms in piperazine each accept a hydrogen bond from the hydroxyl group of two neighboring CBN molecules. There is a third hydrogen bond, where N50 also acts as a donor, forming a hydrogen bond to a third molecule of CBN through an NH···O (ether) interaction. These molecular subunits are further arranged in the crystal packing to form a zigzag chain running along axis c. The calculated crystal shape (Figure 4b) shows a noncentrosymmetric crystal shape with two different parallel largest facets, (100) and (−100), which together comprise 42.15% of the total crystal surface area. The calculated lattice energy is −119.7 kJ mol^–1^. The strongest interaction energies (Figure SI 7c) align with the hydrogen bonding direction, with values of approximately −58.7 and −56.5 kJ mol^–1^.

**Figure 4:**
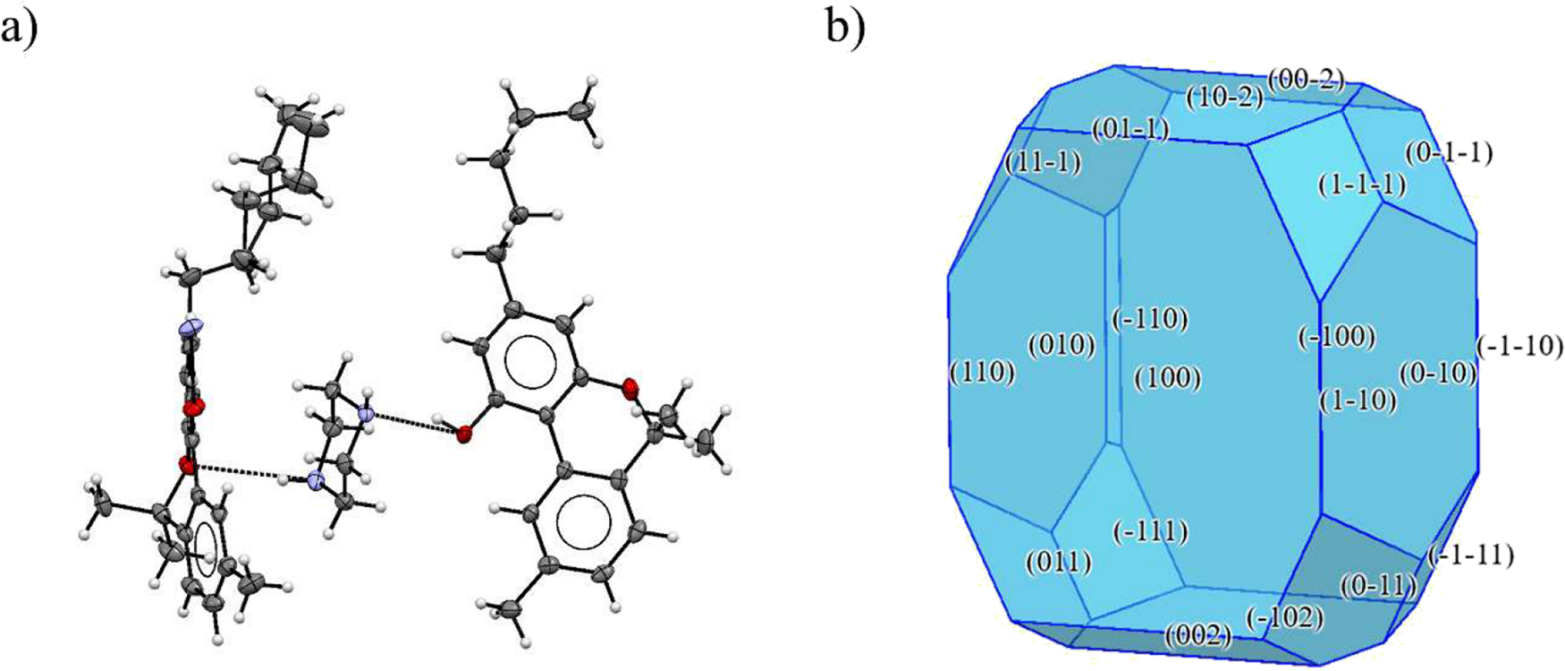
Crystal structure of CBN-PI Form I: (a) unit cell and (b) calculated crystal shape.

#### 3.3.3 CBN-PI Form II

The polymorphic Form II of the CBN-PI cocrystal crystallizes in the orthorhombic system with space group *P b c n*. The asymmetric unit (Figure 5a) comprises one CBN and a half PI molecule. The unit cell (Figure SI 8a) contains eight CBN and four PI molecules. Each piperazine N accepts a hydrogen bond (Figure SI 8b) from a hydroxyl group of a neighboring CBN, creating a centrosymmetric trimer. Weak C–H···O hydrogen bonds (C2a–H2C2a···O2 with C···O ∼ 3.60 Å and H···O ∼ 2.70 Å) link these trimers into columns extended along axis *c*. The calculated crystal shape (Figure 5b) displays a crystal shape dominated by two identical largest facets, (200) and (−200), with an area of 51.64% of the crystal surface. The lattice energy was calculated to be −118.2 kJ mol^–1^. Interestingly, the strongest calculated interaction (−30.1 kJ mol^–1^) occurs between two CBN molecules, which creates the chain motif, while the PI molecule is bonded (Figure SI 8c) in the hydrogen bond direction with an energy of −29.1 kJ mol^–1^.

**Figure 5:**
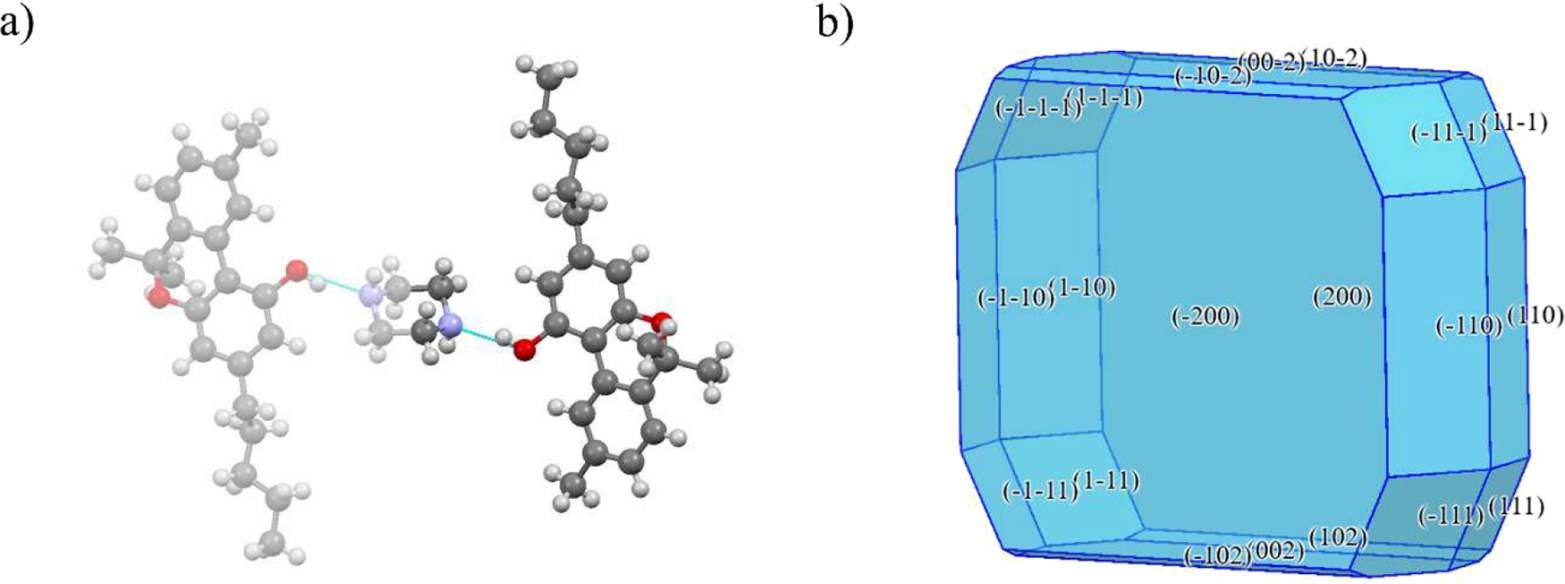
Crystal structure of CBN-PI Form II: (a) unit cell and (b) calculated crystal shape.

#### 3.3.4 CBN-PI Form III

CBN-PI Form III has a monoclinic system with space group *P* 2_1_/*n*. In its asymmetric unit (Figure 6a), it has two molecules of CBN and one molecule of PI. The unit cell (Figure SI 9a) consists of eight CBN and four PI molecules. Each piperazine N accepts a hydrogen bond (Figure SI 9b) from a hydroxyl group of a neighboring CBN, creating a pseudocentrosymmetric trimer.

**Figure 6:**
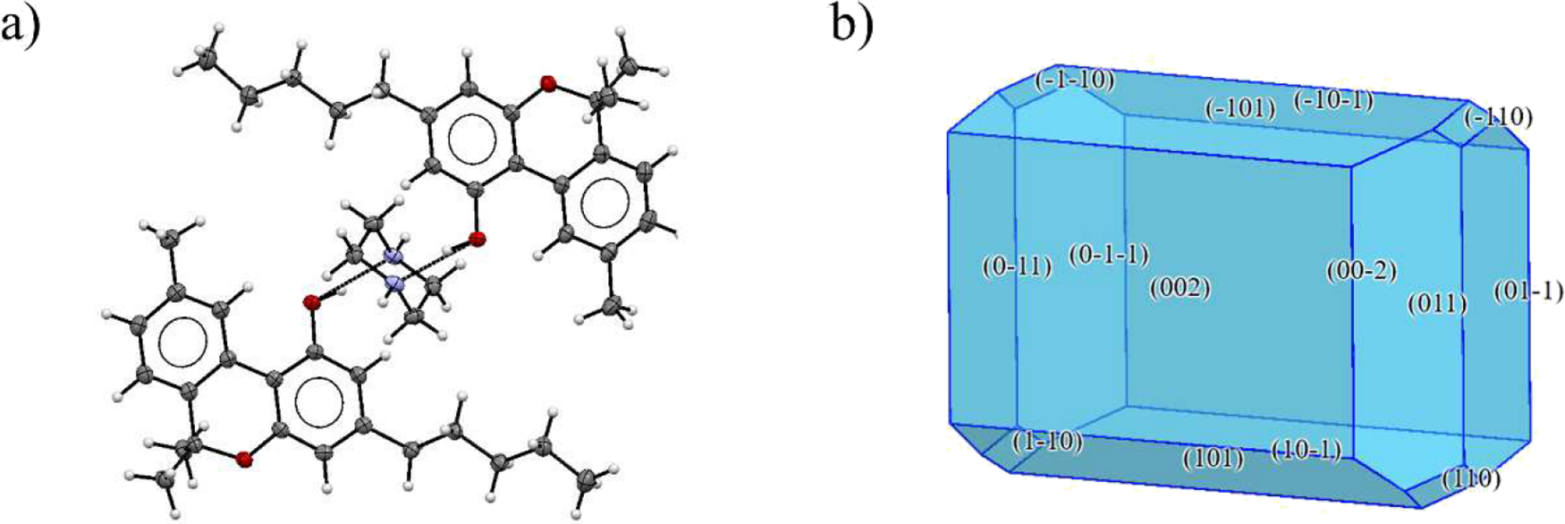
Crystal structure of CBN-PI Form III: (a) unit cell and (b) calculated crystal shape.

Weak C–H···O hydrogen bonds (C49–H491···O24 and C52–H521···O1 with C···O < 3.52 Å and H···O < 2.67 Å) link the trimers into layers parallel to the *ab* plane. The calculated crystal shape (Figure 6b) shows that the largest facets are (002) and (00–2), covering 41.58% of the crystal surface. The lattice energy was determined to be −122.9 kJ mol^–1^. The strongest calculated interactions (Figure SI 9c) occur between the CBN and PI molecules (approximately −69.4 kJ mol^–1^) in the hydrogen bond direction. Furthermore, the CBN molecules between the layers within the chain are slightly weaker (−58.6 kJ mol^–1^).

In summary, Form I exhibits a distinct packing pattern compared to Forms II and III, which probably contributes to its bulkier crystal morphology compared to the plate-like shapes of Forms II and III. In Forms II and III, the hydrogen bond motif is consistent with subunits being arranged into long chains, which create natural spaces corresponding to the slip planes. In contrast, the hydrogen bond system of Form I is arranged as an infinite chain, with one PI molecule connecting three CBN molecules. Interestingly, the Form III exhibits the highest lattice energy (−122.9 kJ mol^–1^) among all polymorphs. Nevertheless, all three forms showed lower lattice energy values than pure CBN (−180.0 kJ mol^–1^). The observed trend in lattice energy is in accordance with the calculated interaction energies, where Form III again shows the highest interaction energy (−69.4 kJ mol^–1^) among all polymorphs, even though it has the lowest melting point (87 °C). Furthermore, its interaction energy is higher than that of pure CBN, which indicates that the thermal properties alone are not the sole determining factor for polymorph stability.^55,57^ Molecular packing efficiency, crystal symmetry, interaction energies, etc. also contribute to the stability difference among the solid-state forms. This is further supported by the unit cell volumes at room temperature (Table SI 5), which revealed that Form III possesses the smallest volume, corresponding to the highest crystal density and closest packing, consistent with its experimentally observed high stability.

### 3.4 Polymorph Comparison

#### 3.4.1 Surface Morphology Analysis

To investigate morphological factors influencing the polymorphic behavior of CBN–PI, we performed surface analyses using CSD-Particle tools. The comparison of the functional groups displayed on the largest facets is shown in Figure 7a, and they are visualized in Figure 8. The functional group density analysis revealed that HB acceptors were present on the surface of Form I (0.014 counts/Å^2^) and Form II (0.012 counts/Å^2^), while HB donors were only found on the surface of Form I, and they are located at the same position as HB acceptors, which correspond to the −OH groups on the CBN molecule. The Form III does not exhibit with any HB donors and acceptors on the studied surfaces. The aromatic bonds were identified on the surfaces of all polymorphs with a higher density than HB acceptors and donors. This might be caused by the presence of two aromatic rings in each CBN molecule, which, when they are oriented parallel to the surface plane, terminate at the crystal surface.

**Figure 7:**
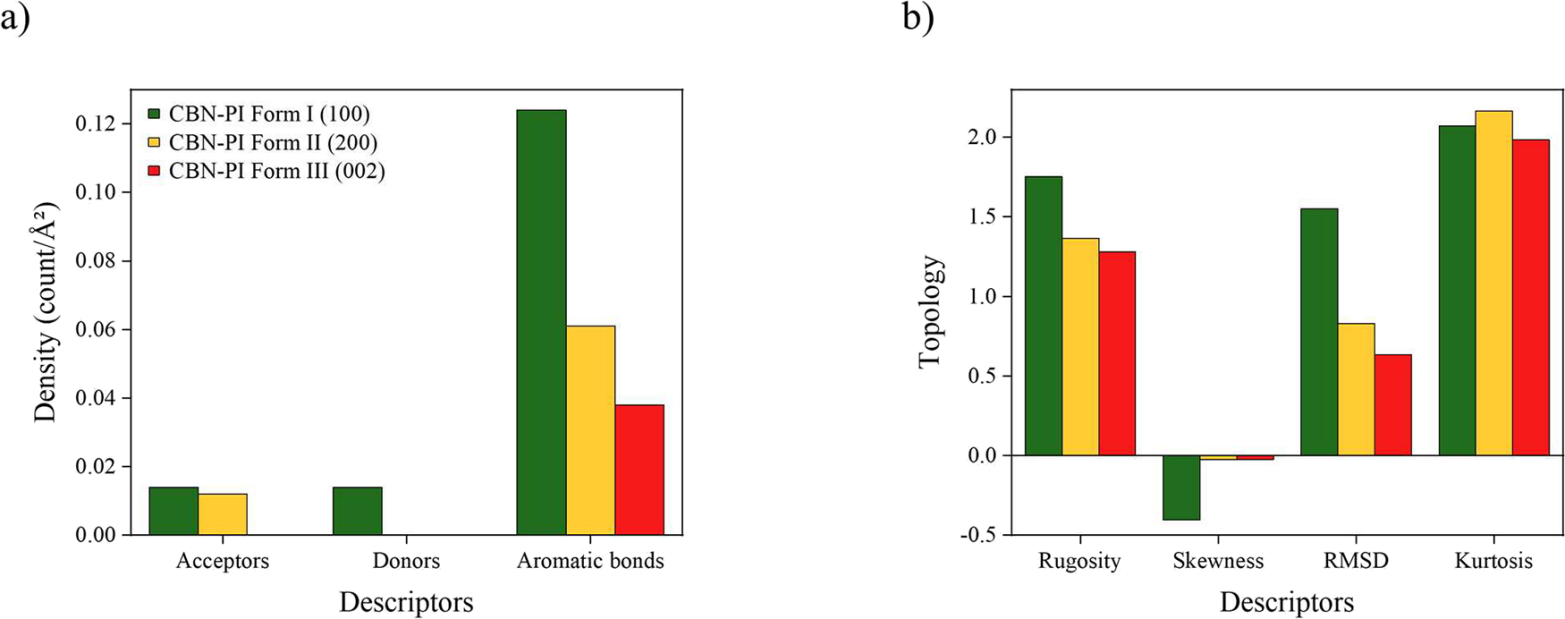
Comparison of the polymorphic forms: (a) density of functional groups displayed on the surface and (b) topology information on the facets.

**Figure 8:**
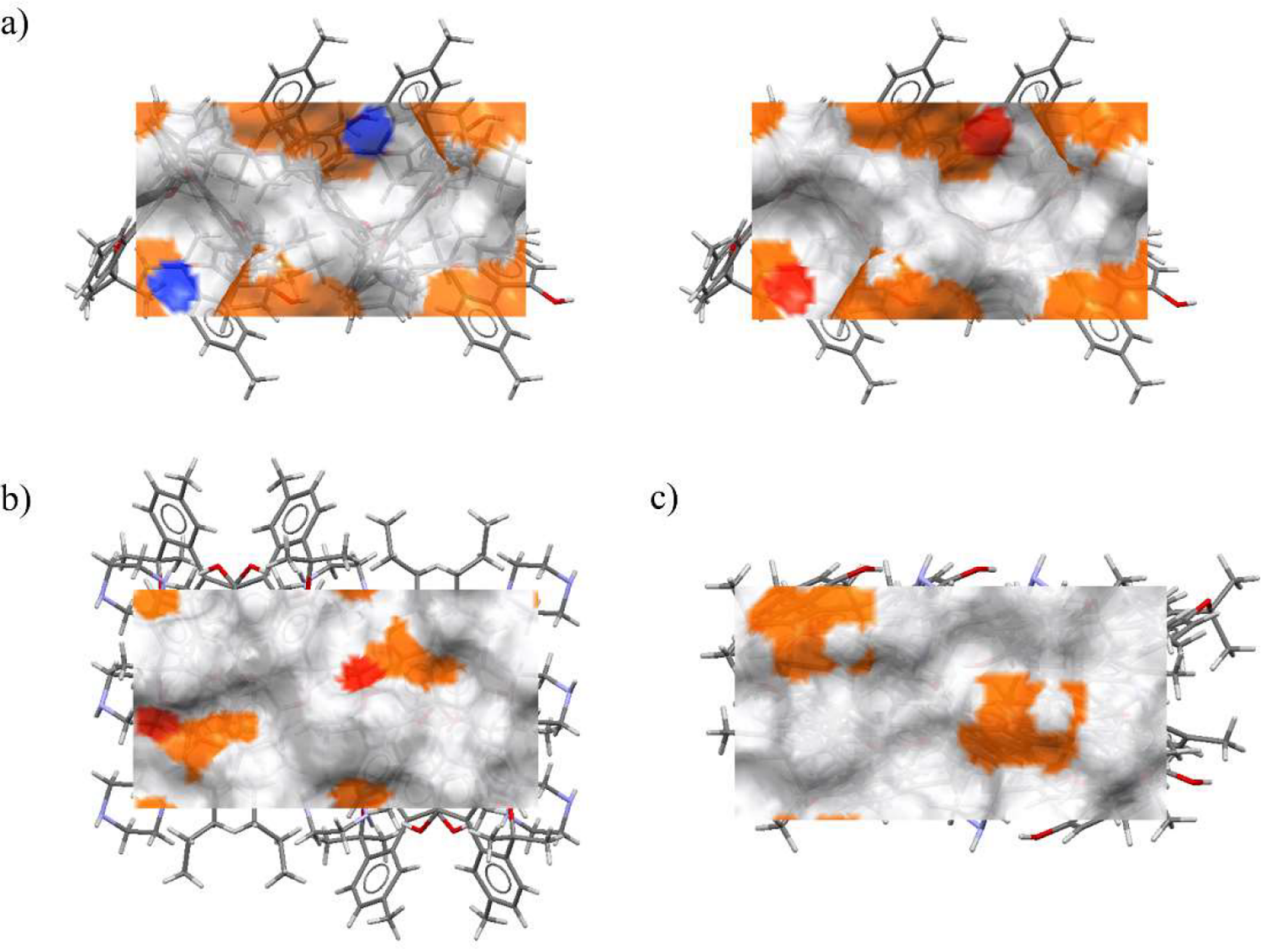
Surface representation of the largest facets of (a) Form I, (b) Form II, and (c) Form III showing different surface properties. Locations of hydrogen bond acceptors (red), hydrogen bond donors (blue), and aromatic bonds (orange). The facet of Form I is doubled in order to show HB acceptors and donors, which are placed in the same positions on the surface.

The topography descriptors, presented in Figure 7b and visualized in Figure SI 10, indicate that Form I exhibits the highest rugosity (1.752), which reflects a rougher surface. Forms II and III exhibit a smoother surface, with rugosity 1.366 and 1.281, respectively. The RMSD (root mean square difference) values follow the same trend observed for rugosity. The skewness value was negative for all forms, which indicates that the height distribution is found below the mean plane. However, the skewness value for Forms II and III was close to zero, meaning the surface is flatter. The kurtosis value was below 3 for all polymorphs, which means the absence of significant hills or valleys.^49^

Based on these obtained results, Form I appears to have the roughest and most chemically interactive surface due to the presence of both HB donors and acceptors, which may enhance its reactivity and facilitate transformation under milling. In contrast, Form III exhibits a smoother and less interactive surface, making it the most stable polymorph from a morphological perspective.

#### 3.4.2 Hydrogen Bond Propensity and Interaction Mapping

To complement morphological insights, hydrogen bond propensity (HBP) calculations and full interaction maps (FIMs) were used to evaluate the statistical favorability of the hydrogen bonding networks inside the crystal structure of each polymorph. The hydrogen bond propensity landscape (Figure 9) plots the mean hydrogen bond propensity on the horizontal axis against the mean hydrogen bond coordination on the vertical axis. Each point corresponds to one of the top-scoring predicted hydrogen bond networks (smaller triangles) or an experimentally observed polymorph structure (large symbol). Networks clustering toward the lower right corner are predicted to be the most favorable in the solid state. The experimentally observed forms are highlighted.

**Figure 9:**
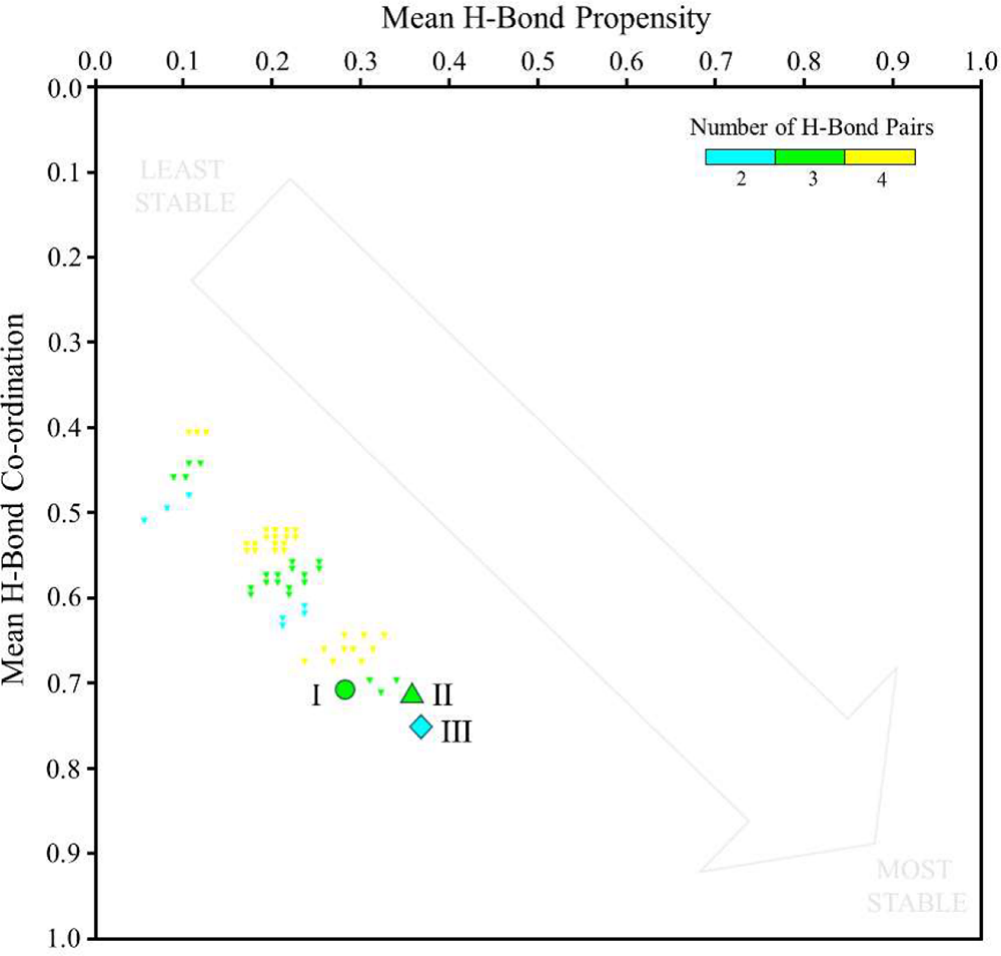
Hydrogen bond propensity landscape of CBN-PI polymorphs.

Across the three polymorphs, Form III has the highest values on both axes (HBP = 0.363, HBC = 0.737), suggesting the best use of available donor–acceptor groups within the crystal structure. Form II (0.350, 0.714) and Form I (0.275, 0.710) rank lower.

Form III contains two intermolecular hydrogen bond pairs, whereas Forms I and II each present three. The greater stability of Form III arises from the quality rather than the number of contacts. In Form III, both CBN phenolic O–H donors address piperazine N acceptors, which is consistent with high likelihood usage for these functional groups. By contrast, Form I introduces a piperazine N–H···O (cycloether) contact, which is statistically less probable and geometrically less favorable. Form II is structurally closer to Form III, but its donor–acceptor vectors are less well aligned. FIM analysis (Figure SI 11) further supports these observations. In Form III, donor and acceptor hotspots align well with actual interactions. In Form I, cycloether O lies in a region of weak acceptor probability, while in Form II, displaced acceptor hotspots lead to geometrical compromises that reduce network quality.

Overall, Form III achieves a “network-economical” structure that leverages strong, accessible hydrogen bonding motifs without introducing weak or frustrated interactions. This likely explains why Form III becomes the dominant product after repeated milling, while Form I disappears. Notably, the most probable NH···N (piperazine) homosynthon is absent in all polymorphs, explaining the modest absolute HBP values but reinforcing the relative advantage of Form III.

In conclusion, comparative analysis of the morphology and hydrogen bonding networks reveals distinct characteristics across the three CBN-PI polymorphs. Form I appears to have the most chemically reactive surface and exhibits the highest surface rugosity and a unique NH···O (ether) interaction, which is statistically weak. This higher reactivity may influence its tendency to disappear upon repeated milling. Form II shows intermediate characteristics, with a smoother surface than Form I but some geometric misalignment in its hydrogen bond contacts, which may limit its stability and allow it to be stable only in a specific mixture, e.g., with Form III. In contrast, Form III exhibits the smoothest surface morphology with no HB donors and acceptors available on the largest surface and the simplest, most favorable hydrogen bonding network. These structural and interaction advantages contribute to its stability and probably explain why Form III becomes the dominant polymorph under the studied conditions.

### 3.5 Particle Energy Calculation

As mentioned above, the stability of the polymorphs is a complex phenomenon, which depends on multiple factors, such as interaction energy, lattice energy, melting points, arrangement in the crystal lattice, etc. To further explore these contributions, we performed DFT-based calculations of the particle energy of the CBN-PI polymorphs. This approach incorporates three key energetic components: lattice energy, conformational energy penalty, and surface energy of the particle. This calculation approach estimates the total energy of a finite crystal particle, thereby providing deeper insights into the solid form stability and enabling the assessment of which contribution plays a dominant role in stabilizing each polymorph. This value was then scaled by the number of formula units per unit cell (Z) to obtain the particle energy per unit cell. The calculated values of each contribution, together with the resulting total particle energy for all polymorphs, are shown in Table 4.

**Table 4:** Calculated Energetic Contributions for the CBN-PI Polymorph Particle Energy

	*E*_particle_ (kJ mol^–1^)	*E*_particle_ per unit cell (kJ mol^–1^)	*E*_latt_ (kJ mol^–1^)	Δ*E*_conf_ (kJ mol^–1^)	0.5∑*x*_(*hkl*)_ × *E*_(*hkl*)_ (kJ mol^–1^)
Form I	–88.8	–355.3	–119.7	2.4	–28.5
Form II	–99.5	–796.2	–118.2	12.8	–6.5
Form III	–113.0	–903.8	–122.9	4.3	–5.7

The lattice energy contribution has already been discussed above (Section 3.3), and it was found that the lattice energy differs only in a few kJ mol^–1^ and From III (−122.9 kJ mol^–1^) showed the highest one. The conformational energy penalty was highest for Form II (12.8 kJ mol^–1^), which means that the molecules must undergo significant distortion from their lowest energy conformation (under vacuum) in order to accommodate the crystal lattice. Conversely, Form I exhibits the lowest conformational energy penalty (2.4 kJ mol^–1^), suggesting the molecular conformation within the crystal is close to the relaxed state in vacuum. Concerning the surface energy penalty, Form I shows the highest value (−28.5 kJ mol^–1^), which means that the surfaces grow faster, producing smaller facets, which are more reactive and result in bulkier crystals,^60^ which is in accordance with the calculated crystal shape (Figure 4b). In contrast, Form II (−6.5 kJ mol^–1^) and Form III (−5.7 kJ mol^–1^) exhibit much lower attachment energy, corresponding to slower facet growth, with less reactive facets and resulting usually in the plate-like morphologies^60^ (Figures 5b and 6b). Finally, the total energy of the particles correlates well with the observed stability of the polymorphs. Form I, with the lowest total energy (−88.8 kJ mol^–1^), was found to disappear completely under the experimental conditions. Form II, with a total particle energy (−99.5 kJ mol^–1^), appeared only under specific conditions along with Form III or with unconverted starting materials. Form III, with the highest total particle energy (−113.0 kJ mol^–1^), proves to be the most stable polymorph. These findings complement the structural and morphological analyses and confirm that Form III is the most stable under the studied conditions.

## 4 Conclusions

In this study, we successfully prepared and characterized a novel cannabinol (CBN) piperazine cocrystal, which exists in three distinct polymorphic forms. By using ball milling, we systematically investigated the influence of various parameters such as the choice of solvents, temperature, and milling time on selective polymorph formation. However, repeated milling experiments revealed the phenomenon of disappearing polymorphs. While Form I was observed at the starting experiments, later on Form III emerged as a dominant phase under nearly all tested conditions. To understand the phenomenon, we further investigated the stability relationship between the polymorphs by performing detailed structural analysis, including crystal packing, hydrogen bond motifs, and morphology. Form I showed a bulkier morphology, with a more reactive surface, whereas Forms II and III exhibited a plate-like morphology with a less reactive surface. Among them, Form III exhibited the highest values of interaction energies among all polymorphs, indicating its superior stability. Furthermore, hydrogen bond propensity and full interaction maps (FIMs) identified that Form III displayed the most favorable, directional hydrogen bonds between CBN hydroxyl groups and piperazine nitrogen atoms. In contrast, Form I contained a statistically weak NH···O (ether) interaction, contributing to the lower network quality. By calculating particle energies, we observed that Form I, despite its strong lattice energy comparable to Forms II and III, exhibited low overall energy (−88.8 kJ mol^–1^), consistent with its disappearance. Form II showed an intermediate energy value (−99.5 kJ mol^–1^), reflecting its stability only under specific conditions and a combination with Form III or the starting material. Form III had the highest overall energy (−113.0 kJ mol^–1^), confirming its dominant stability under the experimental conditions despite its lowest melting point among the polymorphs. This proves that the melting point is not the sole determining factor of the polymorph stability, and other parameters such as crystalline lattice energy, symmetry, and hydrogen bonding system have to be also considered. Together, all of these findings can explain why Form I disappeared and why Form III prevailed. This study provides valuable insights into the polymorphic behavior of cannabinol piperazine cocrystals and highlights the importance of combining computational and experimental approaches in polymorph research. It also emphasizes the importance of a multifaceted approach in the development of a new solid-state form for pharmaceutical applications.

## Supplementary Material



## Notes

Crystal structure data for 2483132 (CBN-PI Form I), 2483133 (CBN-PI Form II), and 2483134 (CBN-PI Form III); NMR spectra; additional XRPD diffractograms; and morphology analysis (PDF)
