# Supplementary material for: Polymorphism in Cannabinol Piperazine Cocrystals: Structural, Morphological, and Energetic Perspectives
Source: Cryst Growth Des. 2025 Nov 25;25(24):10588–99. doi: 10.1021/acs.cgd.5c01390 (PMC12715752; doi:10.1021/acs.cgd.5c01390)
Supplement: Supplementary file 1 [file cg5c01390_si_001.pdf]

Polymorphism in Cannabinol Piperazine Cocrystals: Structural, Morphological, and Energetic Perspectives

Supplementary Information

Adéla Koryťáková[1], Argyro Chatziadi[1], Jan Rohlíček[2], Eliška Zmeškalová[1,2], Josef Beránek[3], Miroslav Šoóš[1,]*

[1]Department of Chemical Engineering, University of Chemistry and Technology in Prague, Technická 3, 16628, Prague 6, Czech Republic

[2]Institute of Physics of the Czech Academy of Sciences, Na Slovance 2, 182 00, Prague 8, Czech Republic

[3]Zentiva k.s., U Kabelovny 130, 10237, Prague 10, Czech Republic

*corresponding author: miroslav.soos@vscht.cz

SI 1 Single crystals measurement

Table SI 1. Crystallographic data and details of refinement of CBN cocrystals.

	**CBN-PI Form I**	**CBN-PI Form II**	**CBN-PI Form III**
Sample	Single crystal	Powder	Single crystal
Empirical formula	$C_{23}H_{31}NO_2$	$C_{23}H_{31}NO_2$	$C_{23}H_{31}NO_2$
Formula weight	353.5	353.5	353.5
Crystal system	Monoclinic	Orthorhombic	Monoclinic
Space group	*Pc*	*Pbcn*	$P2_1/n$
T (K)	95	293	95
Radiation	Mo *K*α	Cu *K*α	Cu *K*α
a (Å)	15.1120 (6)	26.0635 (3)	15.5904 (2)
b (Å)	8.9097 (2)	9.38376 (16)	8.4550 (1)
c (Å)	16.2345 (6)	17.3922 (2)	30.6045 (5)
α (°)	90	90	90
β (°)	110.231 (4)	90	97.6762 (14)
γ (°)	90	90	90
V ($Å^3$)	2051.01 (13)	4253.66 (10)	3998.04 (10)
Z	4	8	8
Reflns. collected	62786	-	64130
Indep. reflns	5544	-	7929
GOF	1.08	3.6538	0.9596
R_1, wR_2 [I > 2δ(I)]	0.047, 0.137	0.0577, 0.0714	0.0489, 0.1147
R_1, wR_2 (all data)	0.0525, 0.1307	0.0629, 0.0720	0.0403, 0.1070
Δρmax, Δρmin (e Å -3)	0.46, -0.30	0.15, -0.20	0.34, -0.35
R_p, R_{wp}, R_{exp}	-	0.026, 0.035, 0.010	-
CCDC number	2483132	2483133	2483134

Table SI 2 Hydrogen-bond geometry (Å, °) of Form I

D—H⋯*A*	*D*—H	H⋯*A*	*D*⋯*A*	*D*—H⋯*A*
C9—H91⋯O1	0.95	2.22	2.853 (5)	123
O1—H11⋯N53^{i}	0.81	1.94	2.733 (5)	168
C31—H311⋯O41	0.95	2.19	2.796 (5)	121
O41—H411⋯N50	0.81	1.88	2.641 (5)	155
N53—H531⋯O15	0.87	2.43	3.279 (5)	164
N50—H501⋯C33^{ii}	0.84	2.52	3.253 (5)	146

Symmetry codes: (i) *x*, −*y*+1, *z*+1/2; (ii) *x*, −*y*+2, *z*+1/2.

Table SI 3 Hydrogen-bond geometry (Å, °) of Form II

D—H⋯*A*	*D*—H	H⋯*A*	*D*⋯*A*	*D*—H⋯*A*
O3—H1O3⋯N1a	0.824	1.9494	2.720	155.2
C2a^{i} —H2C2a^{i} ⋯O2	0.960	2.703	3.598	155.3

Symmetry codes: (i) *x*, −*y*, *z*+1/2;

Table SI 4 Hydrogen-bond geometry (Å, °) of Form III

D—H⋯*A*	*D*—H	H⋯*A*	*D*⋯*A*	*D*—H⋯*A*
C12—H121⋯O15	0.99	2.17	2.8403 (18)	124
C35—H351⋯O38	0.99	2.17	2.8394 (18)	124
O38—H381⋯N50	0.904 (13)	1.837 (13)	2.7163 (18)	163.7 (15)
O15—H151⋯N47	0.893 (13)	1.834 (13)	2.7047 (18)	164.4 (15)

Table SI 5 Comparison of unit cell parameters at RT (Rietveld fit for Forms I and III)

	**CBN-PI Form I**	**CBN-PI Form II**	**CBN-PI Form III**
a (Å)	15.158 (9)	26.0635 (3)	15.778 (7)
b (Å)	9.051 (3)	9.38376 (16)	8.663 (2)
c (Å)	16.460 (5)	17.3922 (2)	32.350 (29)
α (°)	90.0	90	90
β (°)	109.36 (2)	90	109.80 (4)
γ (°)	90.0	90	90
V ($Å^3$)	2130.71<br>(x 2 = 4261.42)	4253.66	4160.45

The crystal structure of CBN-PI was solved from high-resolution laboratory XRPD data using the direct-space approach as implemented in the program FOX (ref.[1]), with the unit cell parameters determined by Dicvol (ref.[2]) within FOX. The initial model of CBN was taken from the known crystal structure available in the CSD under the refcode *CANNOL01* (ref.[3]). The piperazine molecule was built using ChemSketch (ref.[4]). Rietveld refinement was performed with Jana2020 (ref.[5]), where the structure was refined using bond-length and bond-angle restraints consistent with the initial molecular models. Bond distances and bond angles were restrained to target values with standard uncertainties of 0.001 Å and 0.01°, respectively. Hydrogen atoms were placed in geometrically calculated positions. In the final stage of refinement, all atomic positions, three isotropic ADPs (one for piperazine and two for CBN - one assigned to the pentyl chain and the other to the remainder of the molecule), as well as the profile parameters, were refined. The resulting profile fit is shown in Figure SI 1.

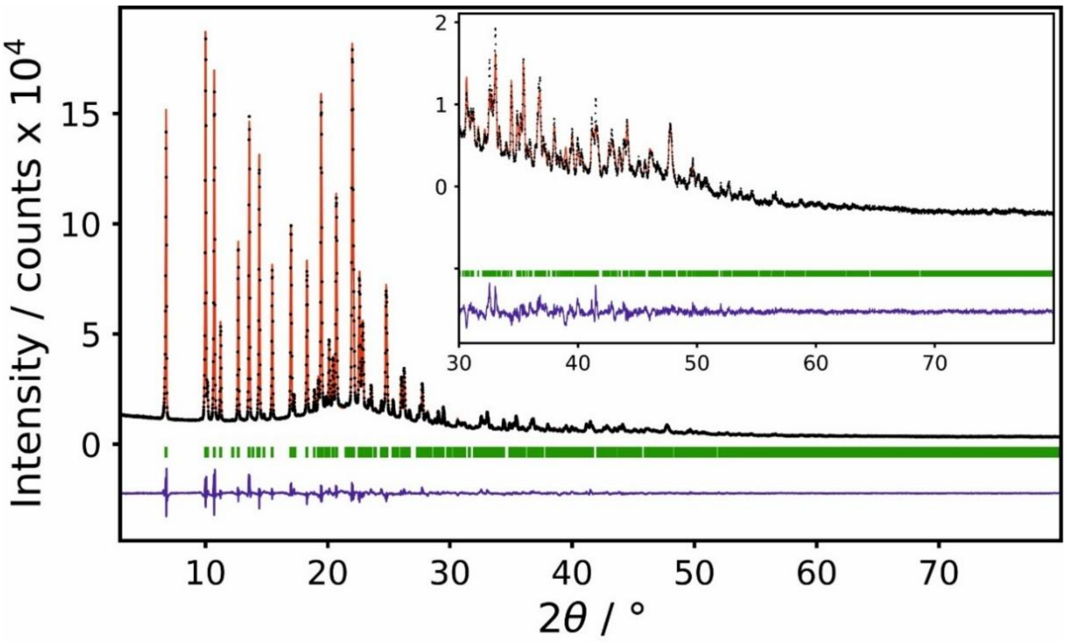

Figure SI 1. The final Rietveld fit of XRPD pattern. Black dots are measured data, red is the calculated profile, blue is the difference between them and green bars are Bragg's positions.

SI 2 Nuclear magnetic resonance

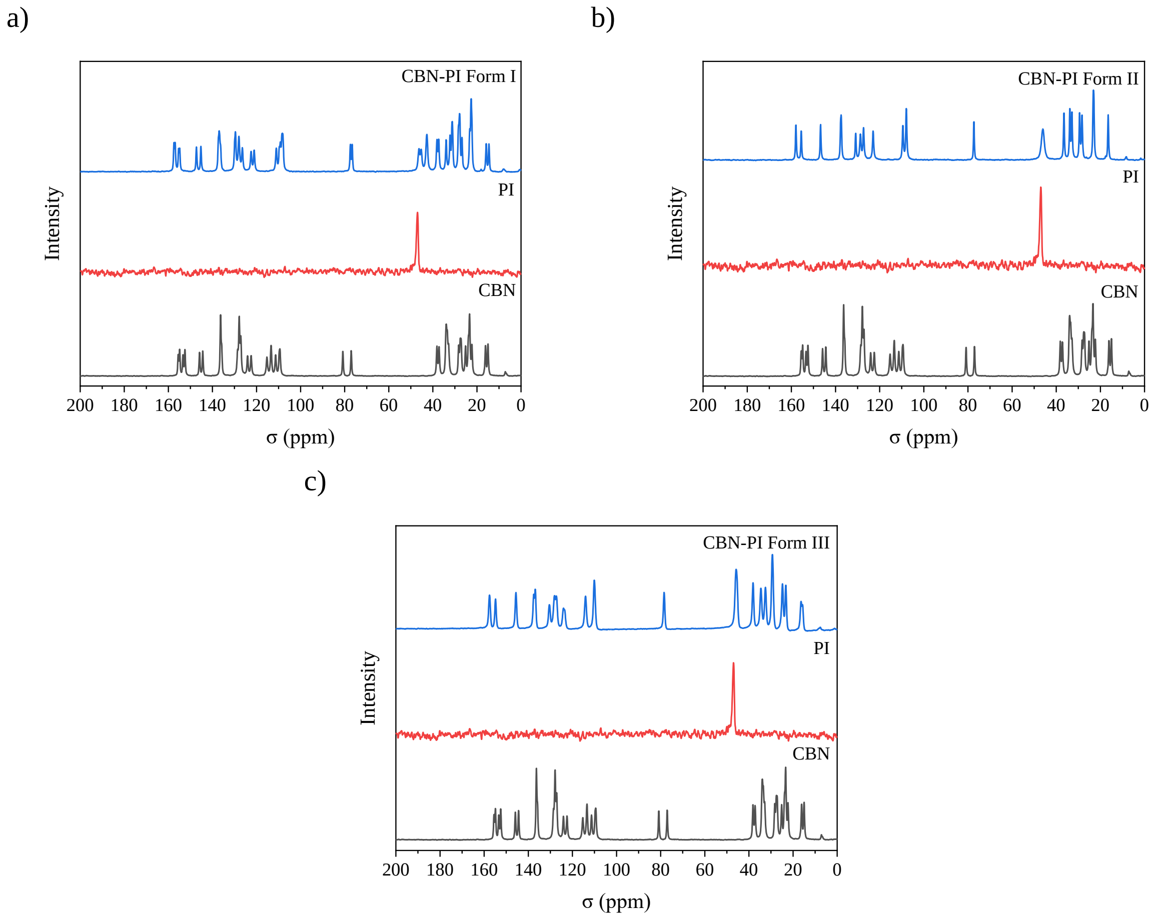

Figure SI 2. Solid-state NMR spectra of cocrystal polymorphs a) CBN-PI Form I, b) CBN-PI Form II, c) CBN-PI Form III.

SI 2 Influence of different experimental conditions on the polymorphic form obtained

Influence of solvent

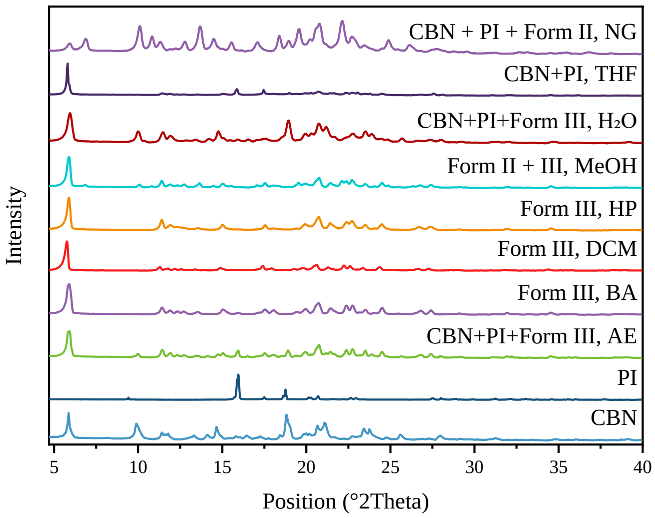

Figure SI 3. XRPD patterns of CBN-PI cocrystal polymorphs obtained using various solvents and neat grinding conditions.

Influence of temperature

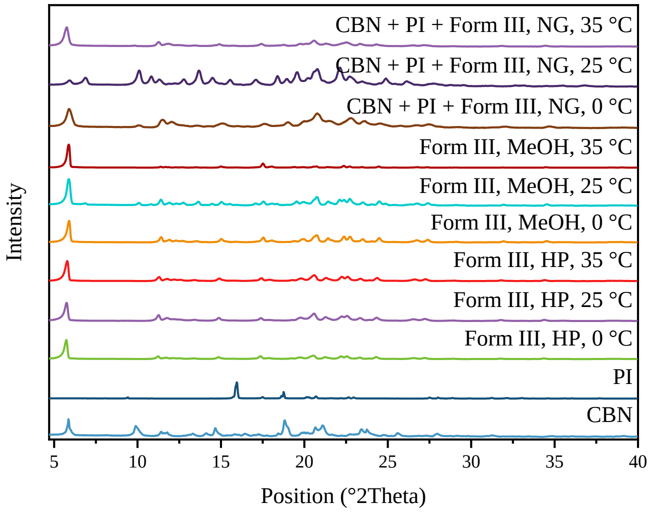

Figure SI 4. XRPD patterns of CBN-PI cocrystal polymorphs obtained at various temperatures using methanol, heptane and neat grinding conditions.

Influence of time

a)

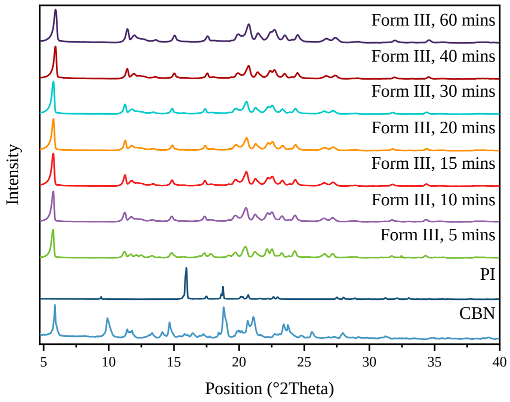

b)

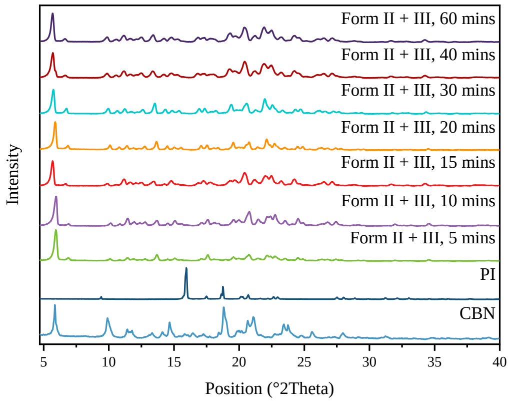

c)

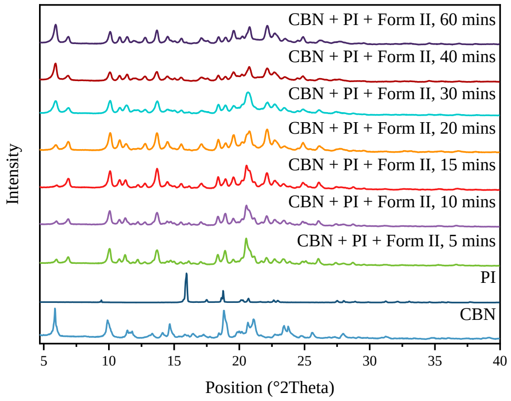

Figure SI 5. XRPD patterns of CBN-PI cocrystal polymorphs obtained at different time points using a) heptane, b) methanol and c) neat grinding conditions.

SI 3 Crystals structures of the solid forms

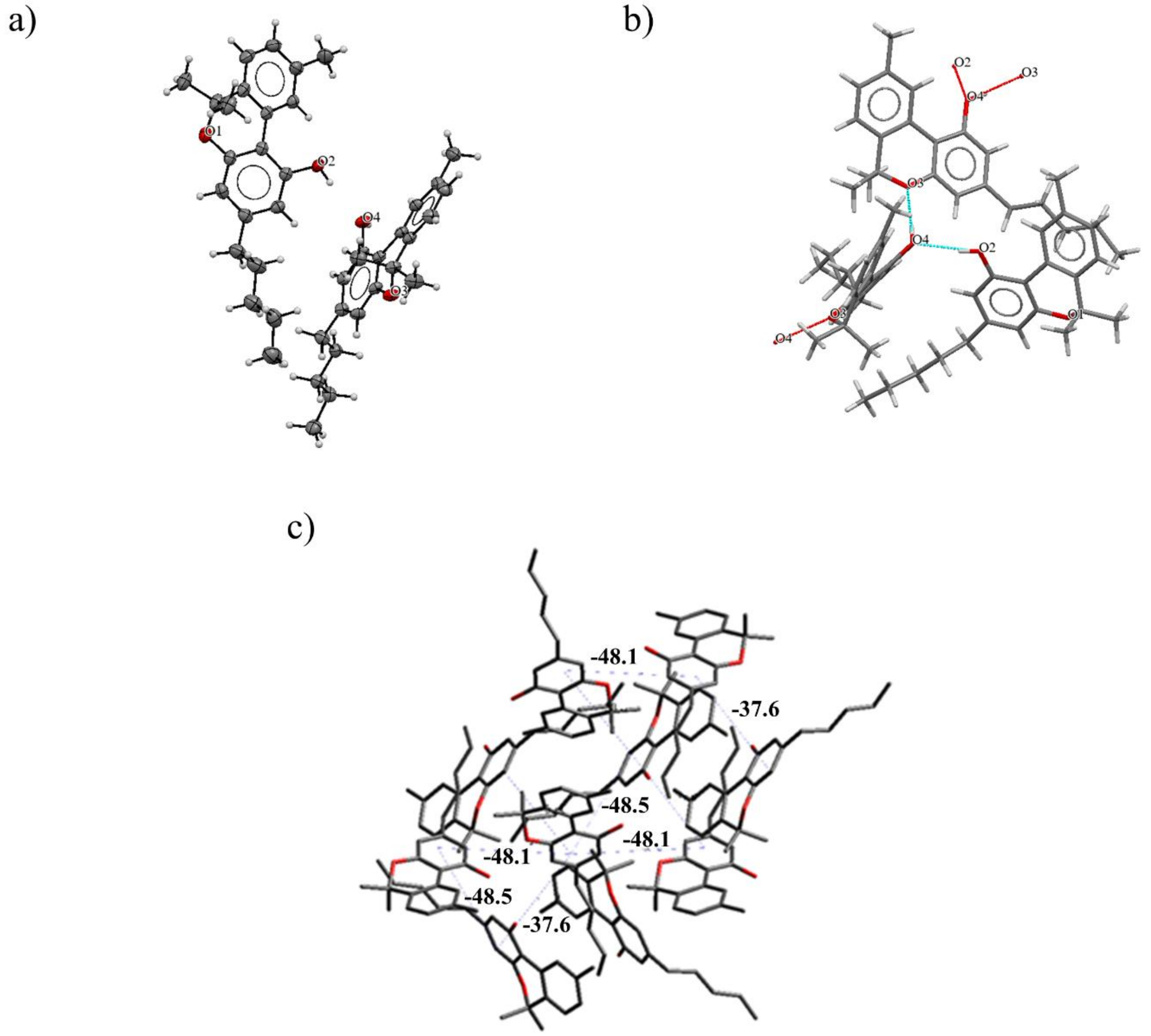

Figure SI 6. Crystal structure of CBN of a) asymmetric unit, b) hydrogen bonding and c) calculated interaction energy.

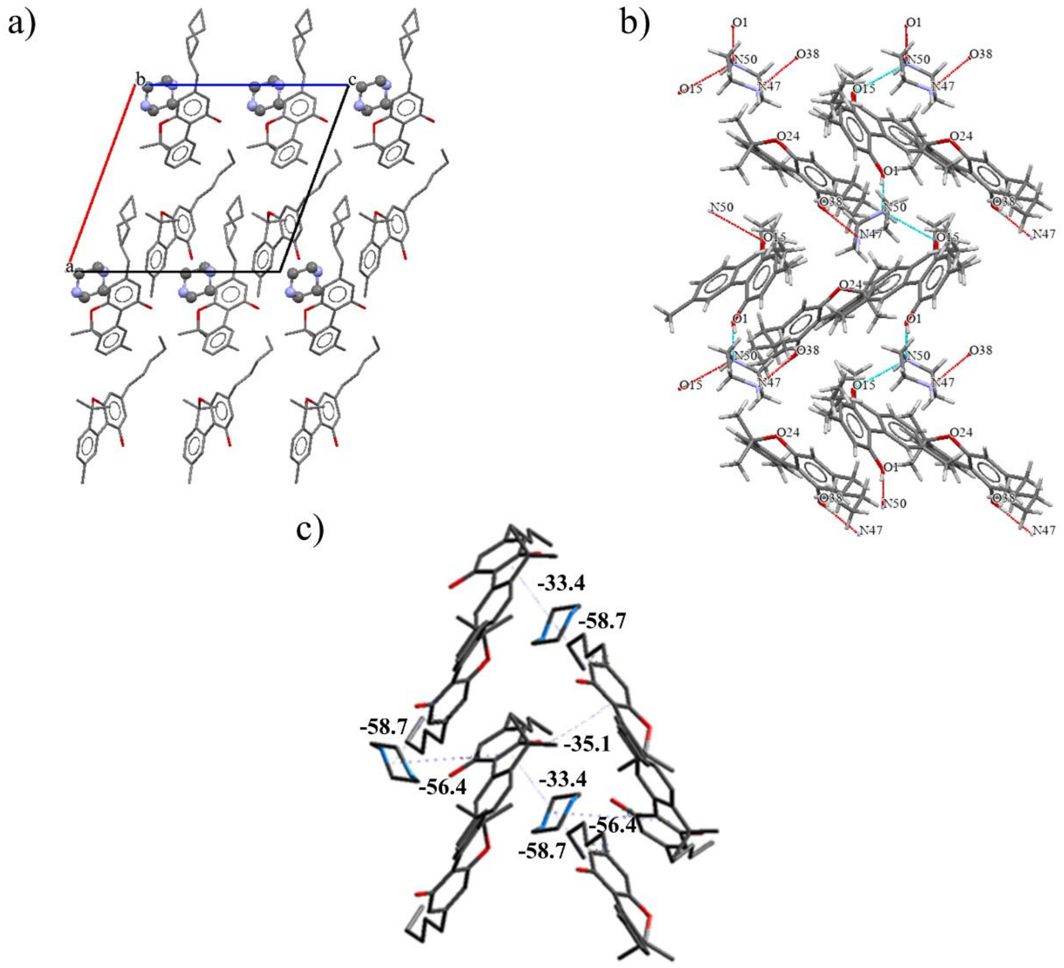

Figure SI 7. Crystal structure of CBN-PI Form I of a) unit cell, b) hydrogen bonding and c) calculated interaction energy.

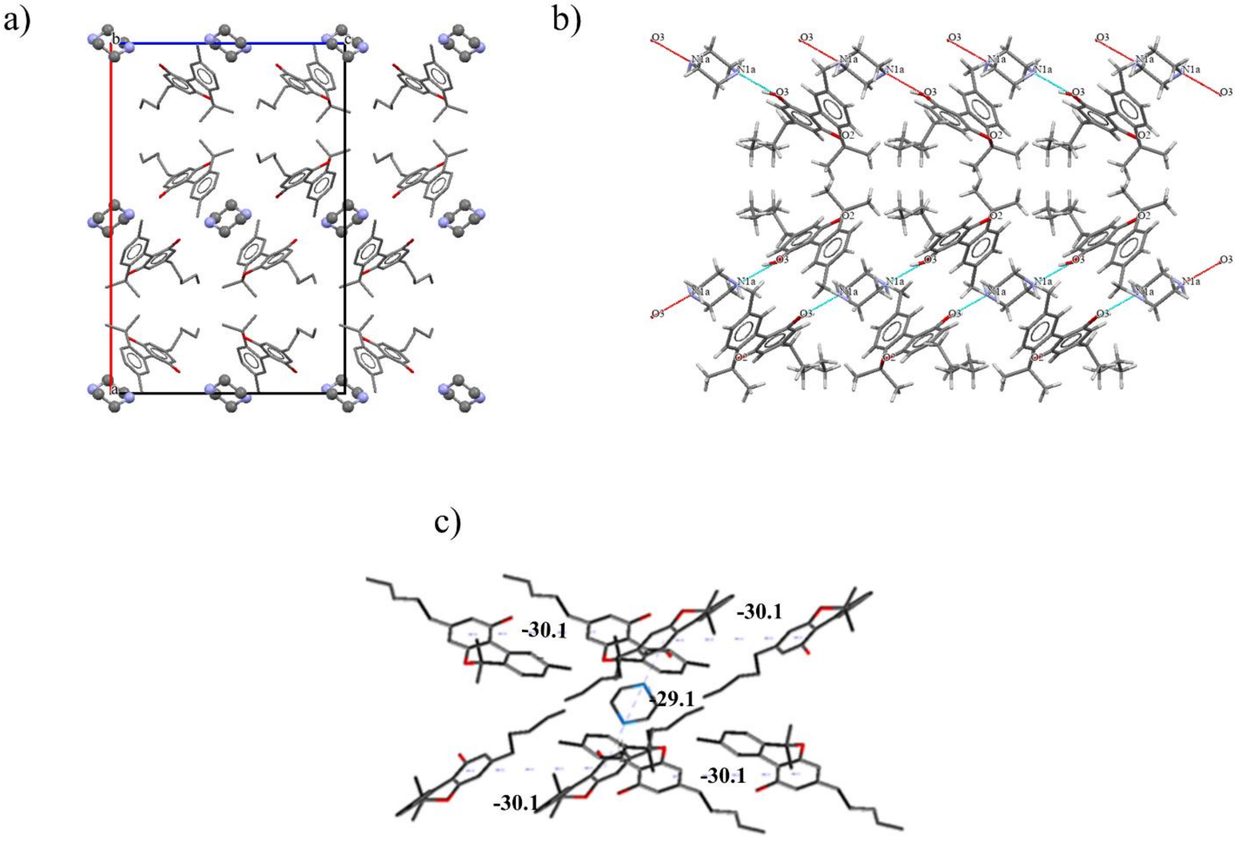

Figure SI 8. Crystal structure of CBN-PI Form II of a) unit cell, b) hydrogen bonding and c) calculated interaction energy.

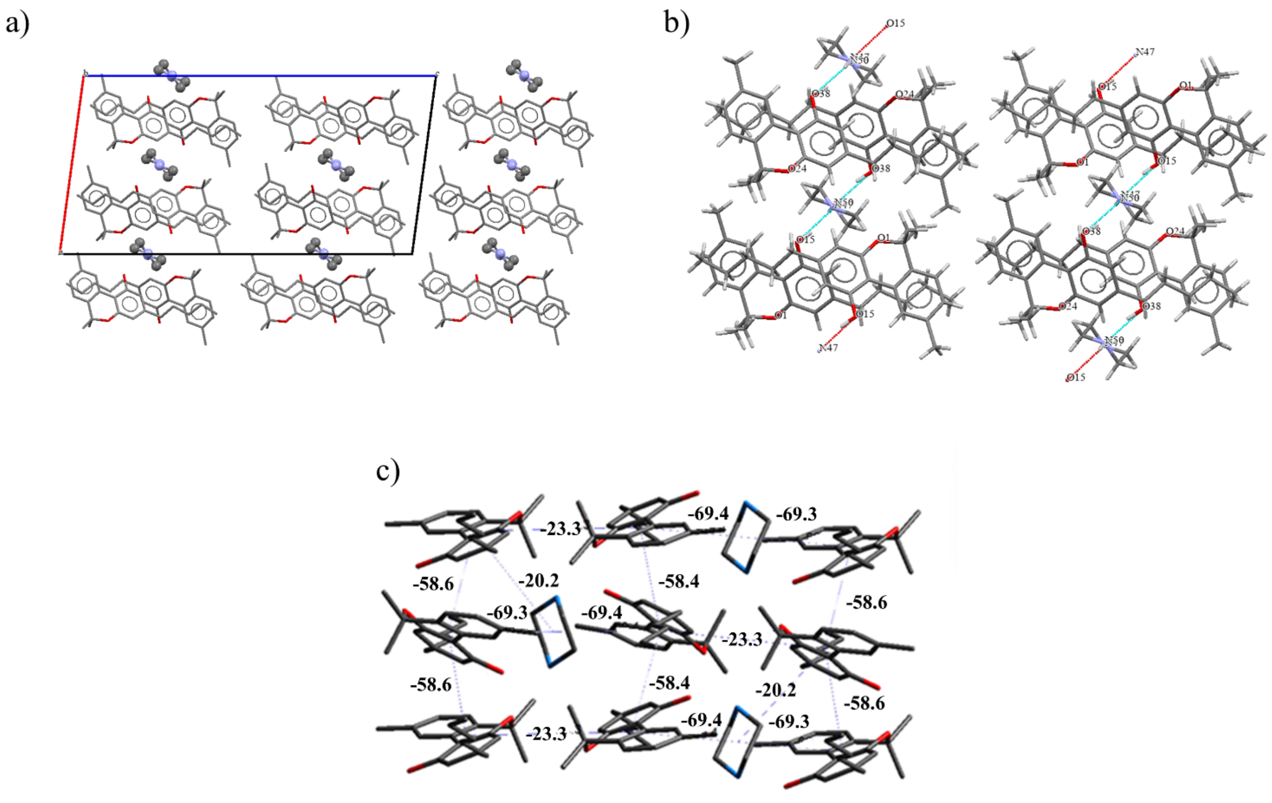

Figure SI 9. Crystal structure of CBN-PI Form III of a) unit cell, b) hydrogen bonding and c) calculated interaction energy.

SI 4 Surface analysis

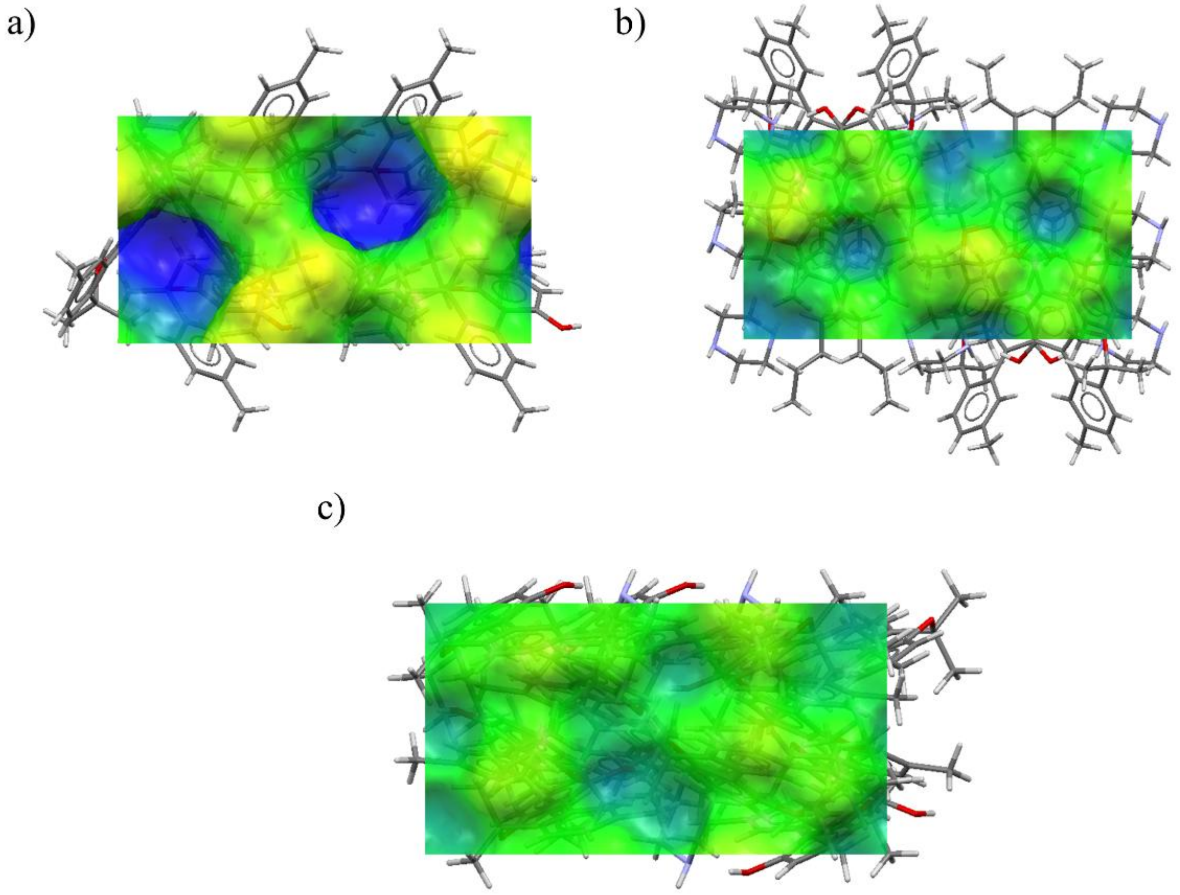

Figure SI 10. A morphology representation of the largest facets of a) Form I, b) Form II, c) Form III showing different surface properties. Location of pits (blue), hills (yellow) and mean plane (green).

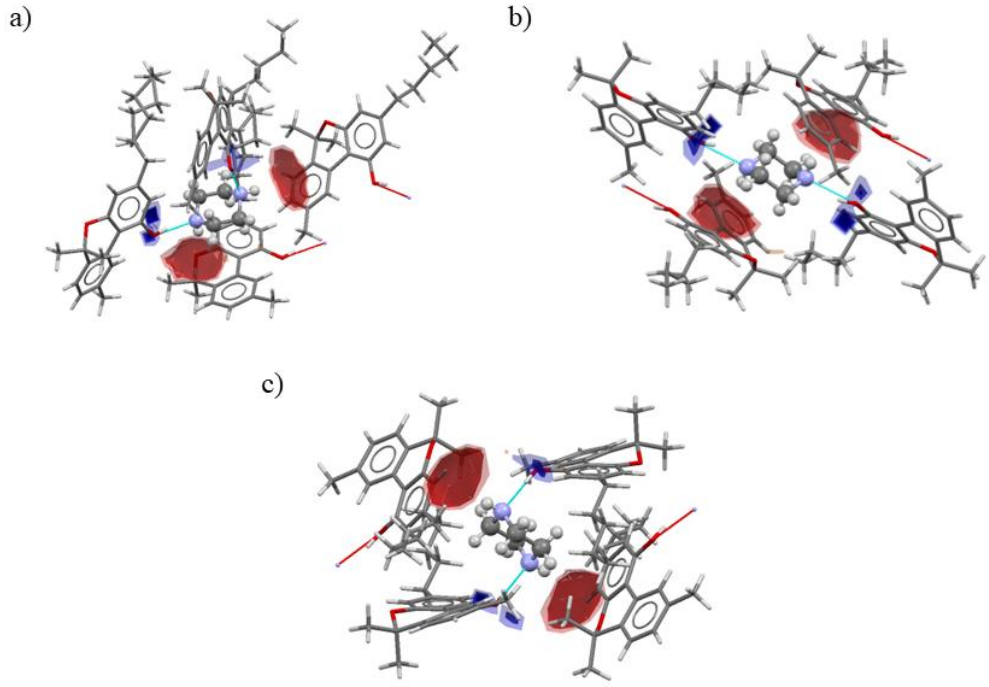

Figure SI 11. Full interaction maps of a) Form I, b) Form II, c) Form III calculated for the piperazine molecule overlaid with the molecules in its vicinity. Red regions show the statistically preferred areas for the presence of an HB acceptor, and blue areas for the presence of HB donors.

Literature

1. V. Favre-Nicolin and R. Cerny, *Journal of Applied Crystallography*, 2002, **35**, 734-743.
2. D. Louër and A. Boultif, *Powder Diffraction*, 2014, **29**, S7-S12.
3. M. R. A. Manap, *Experimental Crystal Structure Determination, CCDC 2217220*, 2022.
4. ACD/Labs, *ChemSketch*, Advanced Chemistry Development, Inc. , Toronto, Canada, 2022.
5. V. Petříček, L. Palatinus, J. Plášil and M. Dušek, *Z. Kristallogr. Cryst. Mater.*, 2023, **238**, 271-282.
